# Ancient use and long-distance transport of the Four Corners Potato (*Solanum jamesii*) across the Colorado Plateau: Implications for early stages of domestication

**DOI:** 10.1371/journal.pone.0335671

**Published:** 2026-01-21

**Authors:** Lisbeth A. Louderback, Cynthia Wilson, Stefania L. Wilks, Kaley Joyce, Sara Rickett, John Bamberg, Alfonso del Rio, Bruce M. Pavlik

**Affiliations:** 1 Department of Anthropology, Natural History Museum of Utah, University of Utah, Salt Lake City, Utah, United States of America; 2 Department of Environmental Science, Policy, and Management, University of California, Berkeley, California, United States of America; 3 USDA/ARS, U.S. Potato Genebank, Sturgeon Bay, Wisconsin, United States of America; 4 Department of Horticulture, University of Wisconsin-Madison, Madison, Wisconsin, United States of America; Universidad de Sevilla, SPAIN

## Abstract

Despite its long history, utilitarian value, and cultural significance to several Indigenous Tribes in the Southwest USA, the extent to which the Four Corners potato (*Solanum jamesii* Torr.) has been domesticated requires circumscription. Establishing the temporal and spatial dimensions of intentional cultivation would provide an essential component of the domestication argument. This project tests the hypothesis that *S. jamesii* tubers were processed with ground stone tools from archaeological sites located beyond the natural range of the species, especially where genetic evidence has previously indicated human transport and establishment in gardens. Microbotanical evidence, in the form of starch granules from 401 ground stone tools at 14 archaeological sites, is examined. More than 6,600 starch granules were recovered from the tools; 163 of which were assigned to *S. jamesii.* Four sites (North Creek Shelter, Long House/Mesa Verde, Pueblo Bonito/Chaco Canyon, and Point of Pines) show consistent use of *S. jamesii* (ubiquity >18%), as early as 10,900 cal BP, and well into Puebloan times. Three of these sites are located far north of the species’ center of distribution in the Mogollon region, across hundreds of kilometers of the Colorado Plateau, and still support an extant population nearby. This suggests an anthropogenic distribution of *S. jamesii* across the Four Corners region and a unique cultural identity around the use of this native potato. These findings, combined with ethnographic interviews and nutritional data, provide clear evidence of use in relation to natural and anthropogenic distributions, thereby allowing an assessment of the degree to which these energy-rich, nutritious, and compact tubers were purposely used and transported.

## Introduction

The dominant paradigm for agricultural origins in the Southwestern, USA is that people adopted exogenous domesticates (i.e., maize, beans and squash) rather than domesticating native plant populations [1]. However, more recent studies have provided compelling evidence that people have been using, cultivating and influencing native plant species over time, including agave [2], barley [3], and amaranth [4]. It is also been established that Indigenous people routinely traded and, therefore, transported high value plant foods across the region throughout the Holocene [5–8]. Such activities would have rapid and drastic impacts on genetic composition of propagules through founder effect alone [9], even without intentional selection for desirable phenotypic traits. Evidence of use, transport and manipulation are, therefore, essential for documenting the initial stages of plant domestication in the Southwestern USA. This study presents evidence of ancient and contemporary use of the Four Corners potato (*Solanum jamesii*) across the Colorado Plateau, corroborating previous work demonstrating long-distance transport and manipulation.

**Fig 1:**
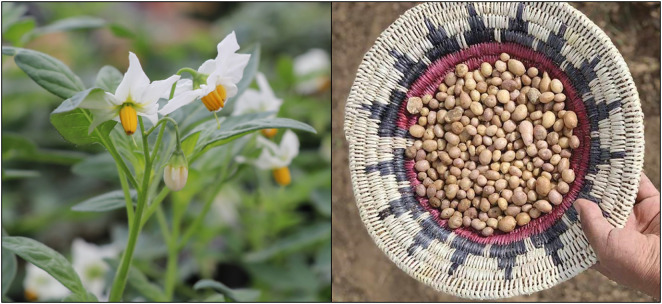
*S. jamesii* plant in flower (left) and tubers in a ceremonial basket (right). Tubers are approximately 1.5–2.5 cm in diameter. Photos by Tim Lee/NHMU and Alastair Lee Bítsoí, respectively.

The tubers of the Four Corners potato (*Solanum jamesii* Torr.) are ideal for transport as they are small and relatively easy to carry while providing starchy nutrition to the carriers (Fig 1). Selecting propagules for transport would invariably lead to reductions in genetic variation (founder effect, a form of genetic drift) and thus initiate manipulation of a once wild food [9–13]. It has also been shown that *S. jamesii* populations associated with archaeological sites lack the ability to sexually reproduce due to human-caused loss of genetic variation [14]. If cultivated, each plant can produce up to 0.6 kg of tubers under optimal conditions during a single growing season. Furthermore, *S. jamesii* tubers can persist underground for at least 14 years [15], achieving high densities and yielding at least 1.7 kg of starchy food per 10^2^ m of habitat area [16]. Thus, a summer-active and highly productive herbaceous perennial could have provided a reliable source of carbohydrate that significantly improved human dietary quality and was available in winter and early spring when other plant foods were not.

The presence of *S. jamesii* growing in atypical habitats among and within archaeological sites across the Colorado Plateau could also be evidence that ancient Indigenous farming practices included this species [16–19]. Therefore, the initial stages (use, transport and manipulation) in the domestication process [10,20–25] that are well-documented in other regions of the world [26] may also have taken place on the Colorado Plateau. Detecting these stages would challenge a long-established scientific paradigm regarding agricultural origins and food choices among Indigenous foragers in western North America by identifying the Colorado Plateau as a hitherto unknown region of plant domestication.

This project tests the hypothesis that *S. jamesii* tubers were processed with ground stone tools at archaeological sites located on the Colorado Plateau and adjacent regions, thus defining the cultural geography of use. Microbotanical evidence in the form of starch granules from 401 ground stone tools at 14 archaeological sites within and beyond the documented range is examined. We predict that tools from archaeological sites within the range of *S. jamesii*, especially sites associated with an extant population, will have a higher frequency of *S. jamesii* starch granules than ground stone tools beyond the range, where there are no extant populations. This analysis, along with ethnographic interviews and nutritional data, will clarify the spatial, temporal and dietary dimensions of tuber use, furthering the argument for domestication of *S. jamesii* in the Southwest USA.

## Materials and methods

### The study species

*Solanum jamesii* is a tuber-forming, diploid species that is widely distributed in central New Mexico and Arizona, with isolated populations extending north into southern Utah and western Colorado (known as the Colorado Plateau of North America) and south into northern Mexico and northwest Texas [27,28]. Of 196 wild potato species (section *Petota*), *S. jamesii* has the fourth largest distribution [27]. Within the center of its distribution across the Mogollon Mountains, *S. jamesii* is closely associated with pinyon-juniper and ponderosa pine woodlands at a mean elevation of 2105 m, but can range between 1415 and 2662 m overall [29].

*S. jamesii* shoots are produced from tubers in early to mid-July following monsoonal rainfall. Flowers appear shortly thereafter. In locations where *S. jamesii* is widely distributed (the Mogollon region of central New Mexico and Arizona), it can reproduce sexually following the cross-pollination of its white flowers (it is an obligate outcrosser), which produce fleshy, green tomato-like fruit. However, vegetative asexual reproduction through its tubers is the far more dominant method of reproduction especially in populations associated with archaeological sites [14]. In southern Utah and Colorado, *S. jamesii* has yet to be observed producing fruit, meaning that these populations are only able to reproduce vegetatively. With ongoing rains, tubers form in late August and September. Although summer droughts and autumn frosts cause the shoots to die off, the tubers remain underground, where they can lie dormant for up to 14 years before sprouting new shoots with additional tubers. As a result, *S. jamesii* tubers are able to persist through years of drought, making them a hardy and versatile food source for the region [15].

**Fig 2:**
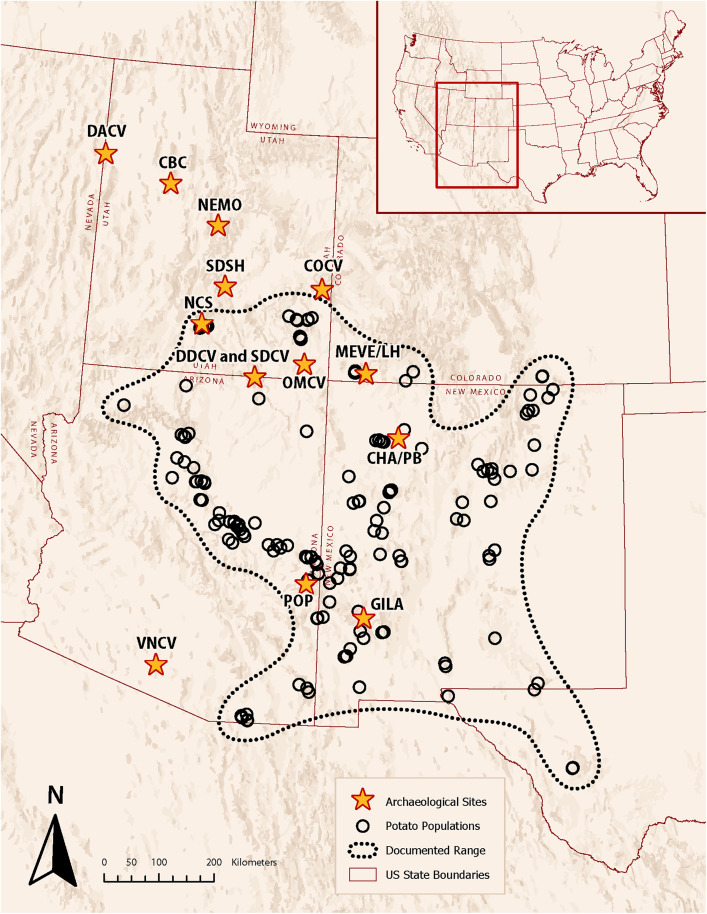
Documented range of *S. jamesii* populations (open circles) [31, with updates]. Locations of the 14 archaeological sites analyzed in the present study are labeled with a star. Site abbreviations are in Table 1. (Basemap is the intellectual property of Esri and is used herein with permission. Copyright © 2025 Esri and its licensors. All rights reserved).

Multiple surveys conducted over the course of 29 years (1992–2021, partly summarized in [30,31]) allow us to document the location and size of extant populations across the entire range of *S. jamesii* (Fig 2; documented locations can be searched in the USDA-ARS Germplasm Resources Information Network – GRIN). We have also observed that some populations occur within 300 m of *in situ* artifacts and with a few exceptions, tend to be small in size (number of aboveground stems between 8 and 500), isolated, at the edge of the species’ distribution, and associated with a wide range of vegetation types (sagebrush scrub, sycamore riparian, oak riparian, and cottonwood riparian in addition to conifer woodlands) [31,32]. Therefore, we define the documented range of this species as simply that which has been confirmed by field surveys and herbarium records, thus including non-archaeological and archaeological populations (Fig 2).

**Table 1:** Archaeological sites, tools, and occurrence of *S. jamesii.*

Archaeological Site	Location	Site Number	Approx. Age Range (cal. BP)	Cultural Period/s	# Ground Stone Tools	Extant *S. jamesii* Stem Counts
Danger Cave (DACV)	Utah	42–13	12,900 − 2,100	Prearchaic – Fremont	16	0
Camels Back Cave (CBC)	Utah	42–392	8,300 - 500	Archaic – Protohistoric	18	0
Nephi Mounds (NEMO)	Utah	42JB2	900	Fremont	16	0
Sudden Shelter (SDSH)	Utah	42SV6	8,700 − 3,600	Archaic	17	0
Cowboy Cave (COCV)	Utah	42WN420	8,800 − 1,300	Archaic – Ancestral Puebloan	16	0
North Creek Shelter (NCS)	Utah	42GA5863	11,300 − 200	Prearchaic – Protohistoric	97	10^2^
Old Man Cave (OMCV)	Utah	42SA1153	8,600 −1,700	Archaic – Ancestral Puebloan	16	0
Long House, Mesa Verde (MEVE)	Colorado	5MV1200	A.D. 1100–1300	Ancestral Puebloan	34	>10^6^
Sand Dune Cave (SDCV)	Utah	NA7523	8,500–1,600	Archaic – Ancestral Puebloan	11	0
Dust Devil Cave (DDCV)	Arizona	NA7613	10,000–1,000	Prearchaic – Ancestral Puebloan	26	0
Pueblo Bonito, Chaco (CHA/PB)	New Mexico	29SJ387	A.D. 800–1200	Ancestral Puebloan	53	10^5^
Point of Pines (POP)	Arizona	AZ W:10:15, W:10:50, W: 9:123	A.D. 1200–1400	Mogollon	28	>10^6^
Gila Cliff Dwellings (GILA)	New Mexico		A.D. 1240–1300	Mogollon	20	10^3^
Ventana Cave (VNCV)	Arizona	AZ Z:12:5	12,300 - 1300	Hohokam	33	0

Characteristics of archaeological sites with available ground stone tools for starch granule analysis. Radiocarbon dates were obtained from CARD and calibrated in OxCal 4.4 with IntCal20 calibration curve. Reported age ranges are maximum and minimum medians. Estimates of local, extant *S. jamesii* stem counts are included, but can have considerable year to year and seasonal variation.

Ethnographic accounts of *S. jamesii* use come from Navajo (Diné), Tewa, Hopi, Apache, Zuni, Kawaik and Zia people. There were various cooking and processing techniques used among the groups including, boiling the potatoes [33–39], grinding/mashing them to make flour [33,40], and/or mixing the potatoes with bentonite clay to reduce bitterness [35–37,39,41,42]. The Apache collected *S. jamesii* in the valleys during late summer. Tubers were “boiled, unpeeled and thus eaten. The *S. jamesii* product was sometimes dried, stored, and later ground into flour for making bread” [33, p. 42]. Pioneers in Escalante, southern Utah also consumed the potato from the 1860’s through the 1930’s [43].

### Sites and tools

There are many archaeological sites within and beyond the documented range of *S. jamesii*. Relatively few have been excavated and fewer still having ground stone tools from buried deposits with published contextual details. Only excavated and recorded sites that had available ground stone tools would be suitable for starch granule analysis. Fortunately, eight repositories had collections from 14 sites that met these criteria and provided a wide temporal range (early Holocene through Protohistoric) for sampling a sufficient number of properly stored tools.

Inventories of ground stone tools (i.e., manos and metates) from the 14 sites were examined to determine the availability and feasibility for sampling. Objects were not chosen if they 1) were on exhibit, 2) have an unknown location at the repository, 3) were not collected, or 4) are restricted objects. Approximately 98% of the ground stone tools sampled contain site and/or stratigraphic provenience. Furthermore, information regarding curation practices (i.e., handling, cleaning, and storage) was considered so that the probability of cross contamination was low (i.e., objects bagged individually, stored in sealed boxes vs. open shelves, etc.). A total of 401 ground stone tools from eight repositories (American Museum of Natural History, Arizona State Museum, Brigham Young University Museum of Peoples and Cultures, Edge of the Cedars, Mesa Verde Visitor and Research Center, Museum of Northern Arizona, Natural History Museum of Utah, and the Western Archaeological Conservation Center) were ultimately sampled for starch granule analysis (S1 Table available on Dryad).

### Starch granule analysis

Starch granules are microscopic plant structures formed from photosynthates by subcellular amyloplasts as energy reserves [44]. Long-term energy reserves, or “storage starches” are most abundant in seeds, fruits, and underground storage organs (USOs - corms, tubers, taproots, etc.). These starch granules are released from cells during food processing and can subsequently become deposited in the cracks and crevices of archaeological tools. Because of their microcrystalline structure, starch granules are relatively resistant to organic decay and are often preserved for millennia in archaeological contexts [45].

Consumable supplies used in this study, such as nitrile gloves, weigh boats, toothbrush heads, and test tubes, were only used once to prevent cross-contamination during sampling and processing. Extraction of starch granules began with placing a portion of each ground stone tool in a weigh boat filled with deionized water (diH_2_0), which was then placed in a sonicator. Stones that were too large to fit in the sonicator were spot sampled with an electric toothbrush. Spot sampling uses a smaller surface area than sonication, so starch yield may be affected (46). Each sample was then filtered with diH_2_0 using a 125 µm U.S.A. standard test sieve and transferred to 50 ml sterile test tube. Samples were centrifuged for three minutes at 3000 RPM. The supernatant was discarded, and the re-suspended sample pellet transferred to a 15 ml sterile test tube.

Heavy liquid was used to isolate starch granules from the samples; 5 ml of lithium heteropolytungstate (LST – specific gravity 2.00–2.35) (Central Chemical Consulting, Malaga, Australia) was added to each sample and resuspended with a vortex mixer. The sample was then centrifuged for 15 minutes at 1000 RPM. The suspended fraction was decanted into another sterile set of labeled test tubes. Two more rinses (addition of diH_2_0 and centrifugation, three minutes at 3000 RPM) removed any residual heavy liquid. Acetone was added, then each sample was spun and allowed to dry overnight. This process resulted in the formation of a small pellet of organic material that would include starch granules, if present.

#### Starch granule identification.

Following processing, pellets were re-suspended in a few drops of a 50/50 glycerol and diH_2_O solution and then mounted on a glass slide. Each slide was scanned in its entirety using a transmitted brightfield microscope fitted with polarizing filters and Nomarski optics (Zeiss Axioscope 2, Zeiss International, Göttingen, Germany). A digital camera (Zeiss Axiocam HRc) with imaging and measurement software (Zen core v2.7) were used to capture images and to make starch granule measurements. Because ground stone tools were made available from repositories over a six-year period, imaging and measurements were performed by multiple collaborators. Consistency was assured by frequent trainings and assigning each collaborator to all tools from a single site.

Previous studies discuss our approach for identifying *S. jamesii* starch granules [17,47,48]. Those studies developed a set of statistically defined diagnostic characteristics that include: 1) possession of an eccentric hilum, 2) the presence of a longitudinal fissure, 3) the absence of fissure branching, 4) a ratio of fissure width to granule width in the range of 0.21–0.28 (mean ratio ± 99% CI), and 5) mean maximum granule length greater than 34.88 μm (mean minus the 99% CI). Archaeological granules that have an eccentric hilum and possess two or more of the characteristics are taxonomically assigned to *S. jamesii* (consistent with [17]). Granules with eccentric hila and possess less than two of the characteristics could be *S. jamesii* but could also belong to other plant taxa with USOs (e.g., Liliaceae) and in this study remain unidentified.

#### Control samples.

Starch occurs naturally on laboratory surfaces and can be airborne [49–51], so precautions were taken to minimize contamination during laboratory processing. Beginning in 2023, passive traps [49] were placed in the NHMU laboratory spaces and left in place for approximately one week during sample processing. Prior to 2023, contamination was routinely managed by wiping down laboratory surfaces with an ethyl alcohol solution.

In addition to passive traps, control samples were taken from feature fill sediments and/or animal bones to detect background levels of starch at four archaeological sites. Sediment control samples from North Creek Shelter were processed and analyzed in 2013 [46]. However, subsequent research has shown that sediments may not be ideal control samples due to natural occurrence of starch and enzymatic damage caused by soil bacteria [52]. Therefore, we sampled animal bones from the same cultural deposits where ground stone tools were recovered at Danger Cave, Camels Back Cave, and Sudden Shelter.

Not all archaeological sites had sediment or faunal bones available for sampling, as many collections were curated decades ago, before the routine practice of collecting associated sediment during excavation. Moreover, submitting additional research requests to sample sediment or faunal material from curated collections was not always feasible. Although feature fill sediments and animal bones located in situ near artifacts may have been exposed to starch from the processing of associated plant materials, the quantity of starch granules from such sources is expected to be minimal compared to starch granules embedded in the cracks and crevices of ground stone tools.

#### Relative abundance and ubiquity.

Relative abundance of *S. jamesii* starch granules for each site was calculated using the following formula: ([number of individual *S. jamesii* starch granules/total number of individuals of all other granules] x 100). It is used to measure abundances of *S. jamesii* across time and space and can inform about the importance of *S. jamesii* to past human diets. Relative abundance was calculated for each site examined in this study.

The ubiquity index (expressed as a percentage) describes the occurrence of *S. jamesii* starch based on the number of ground stone tools from which that resource is recovered: % of *S. jamesii* = (Number of tools with S*. jamesii* starch/Total number of tools) x 100 [53]. Higher percentages indicate more pervasive use, thus having implications for the stability of resource availability as well as cultural importance. This was calculated for each site examined.

### Elder interviews

Interviews with 15 Diné elders, eight female and seven male, were conducted between November 16, 2018 and January 16, 2020 by a team of two – one Diné female (coauthor C. WIlson) as the conversant and one Diné male (Woody Lee) as the interpreter (the spoken language was largely Diné). Informed written consents were obtained for each interviewee. The interviews took place in the home or on the property of the elder. Consistent topics and questions included 1) specific reference to wild potatoes and onions as tuber/bulb foods, 2) identity and use of medicinal plants, 3) crop plants and farming methods, and 4) hunting traditions. Interviewees were also shown tubers of the Four Corners potato to confirm their synonymy with “wild potato”. Other topics informed public land use that supports restoration of traditional foodways [19], firewood collection [54], and cultural continuity of intergenerational knowledge transmission among the informant and interviewees. The interviews were recorded, transcribed and are archived at Utah Diné Bikéyah where the identities of the elders are held *incognito*.

### Nutritional analysis of tubers

Greenhouse-grown tubers of *S. jamesii* from five archaeological populations in southern Utah and southern Colorado (Escalante, Bears Ears National Monument, Newspaper Rock, Mesa Verde Lower Navajo Canyon, and Mesa Verde Spruce Canyon) were sent to NutriData (Laguna Beach, California, www.nutridata.com) for nutritional analyses. The medium was standard potting soil without fertilizer or mineral solutions. Total fresh weight of tuber batches was approximately 550 g, pooled from 2–3 site collections each. In addition, a sample of store-bought organic red potatoes was sent for comparison. Nutridata uses US-FDA Compliant analytical methods for food values (calories, total fat, saturated fat, total carbohydrate, dietary fiber, total sugar, protein), mineral content (Ca, P, K, Mg, Mn Zn, Fe, Na) and a limited panel of vitamins (niacin, total folate, B1-thiamine, B2-riboflavin, D).

## Results

### Sites and tools

In all, 401 ground stone tools were available from 14 archaeological sites that are located across the documented range of *S. jamesii* and beyond (Table 1 and S1 Table). Site deposits included the entire Holocene, some dating as early as 12,900 cal BP and others as late as 200 cal BP. Among these were five sites (North Creek Shelter, Long House/Mesa Verde, Pueblo Bonito/Chaco Canyon, Point of Pines, and Gila Cliff Dwellings) that have extant populations of *S. jamesii* growing within 300 m (sometimes much closer). The size of those populations varies greatly, but all have been documented over 29 years of field survey [31 with updates].

The available ground stone tools have been identified as either manos or metates and were manufactured from sandstone, quartzite, and fine grained volcanic. They ranged in size and weight from small, thin fragments to large, thick trough metates. Approximate ages for the ground stone tools are based on the radiocarbon dates of the deposits that are published in the Canadian Archaeological Radiocarbon Database (CARD, [55]). We calibrated the normalized dates in OxCal 4.4 using the IntCal20 curve [56] and followed chronological interpretation based on published primary research.

### Starch granule analysis

**Fig 3:**
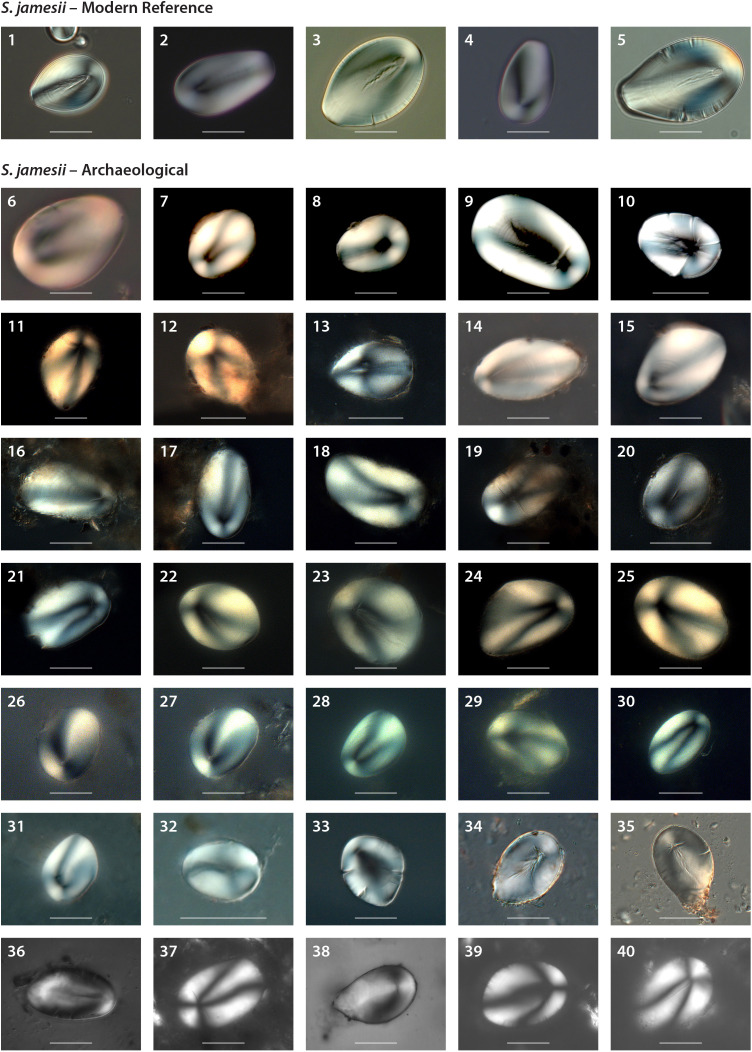
Starch granules extracted from ground stone tools at various archaeological sites assigned to *S. jamesii.* 1-5 = Modern reference material of *S. jamesii* [48], 6 = Nephi Mounds, 7-10 = Sudden Shelter, 11-12 = Dust Devil Cave, 13 = Sand Dune Cave, 14-15 = Ventana Cave, 16-21 = Long House, Mesa Verde, 22-30 = Pueblo Bonito, Chaco Canyon, 31-33 = Point of Pines, 34-40 = North Creek Shelter. Scale bars represent 20µm.

Extractions of interstitial starch residues were performed on all 401 ground stone tools. Although 350 of those samples yielded a total of >6,600 starch granules, 61 ground stone tools bore 163 granules that were assigned to *S. jamesii* (i.e., possessing an eccentric hilum plus two or more diagnostic characters) (Table 2, S2 and S3 Tables available on Dryad). Confidence in taxonomic assignment was substantially increased by the majority of these granules also having a maximum length >35 µm and a narrow, unbranched longitudinal fissure (S3 Table available on Dryad). *S. jamesii* granules came from tools in deposits at nine sites: Nephi Mounds, Sudden Shelter, North Creek Shelter, Mesa Verde, Pueblo Bonito, Dust Devil Cave, Sand Dune Cave, Point of Pines, and Ventana Cave (Fig 3). None were found at the two northernmost Great Basin sites (Danger Cave, Camels Back Cave). A small number of granules did not possess more than two criteria used to identify *S. jamesii* and could have come from other species that have underground storage organs with starch granules having eccentric hila (e.g., Liliaceae). The majority of starch granules observed belong to *Zea mays*, *Pinus* spp., Apiaceae, and grasses (probably Triticeae). Control samples showed significantly fewer granules when compared to the archaeological samples (t(3) = 2.99, p = 0.03) and none of the starch granules from the control samples resembled *S. jamesii*. Passive traps did not detect the presence of any starch granules.

**Table 2:** Starch granule yields and *S. jamesii* relative abundance and ubiquity.

Archaeological Site	# Starch Granules (Tools)	# Unidentified USO	# *S. jamesii* granules	# Tools with *S. jamesii*	*S. jamesii* Relative Abundance	*S. jamesii* Ubiquity	# Starch Granules (Control)
Danger Cave (DACV)	992	6	0	0	0.00	0.00	1
Camels Back Cave (CBC)	616	3	0	0	0.00	0.00	2
Nephi Mounds (NEMO)	285	0	1	1	0.35	6.25	---
Sudden Shelter (SDSH)	375	6	4	2	1.07	11.76	25
Cowboy Cave (COCV)	15	0	0	0	0.00	0.00	---
North Creek Shelter (NCS)	1383	52	70	17	4.33	17.71	9
Old Man Cave (OMCV)	90	0	0	0	0.00	0.00	---
Long House, Mesa Verde (MEVE)	572	18	9	6	1.57	17.65	---
Sand Dune Cave (SDCV)	293	6	1	1	0.34	9.09	---
Dust Devil Cave (DDCV)	391	8	3	1	0.77	3.85	---
Pueblo Bonito, Chaco (CHA/PB)	1222	113	68	26	5.56	49.06	---
Point of Pines (POP)	137	4	5	5	3.65	17.86	---
Gila Cliff Dwellings (GILA)	43	4	0	0	0.00	0.00	---
Ventana Cave (VNCV)	206	8	2	2	0.97	6.06	---
**Totals**	6619	228	163	61			

Total number of starch granules extracted from ground stone tools (n = 401) at 14 archaeological sites, abundant granules from other plant taxa (but not included in this analysis), total number of unidentified USO starch granules (those with eccentric hila and possess less than two of the *S. jamesii* diagnostic criteria), number of starch granules assigned to *S. jamesii*, number of tools from each site with *S. jamesii* granules, relative abundance and ubiquity of *S. jamesii* granules, and total number of starch granules extracted from control samples from each site.

### Geographic occurrence of *S. jamesii* starch granules

**Fig 4:**
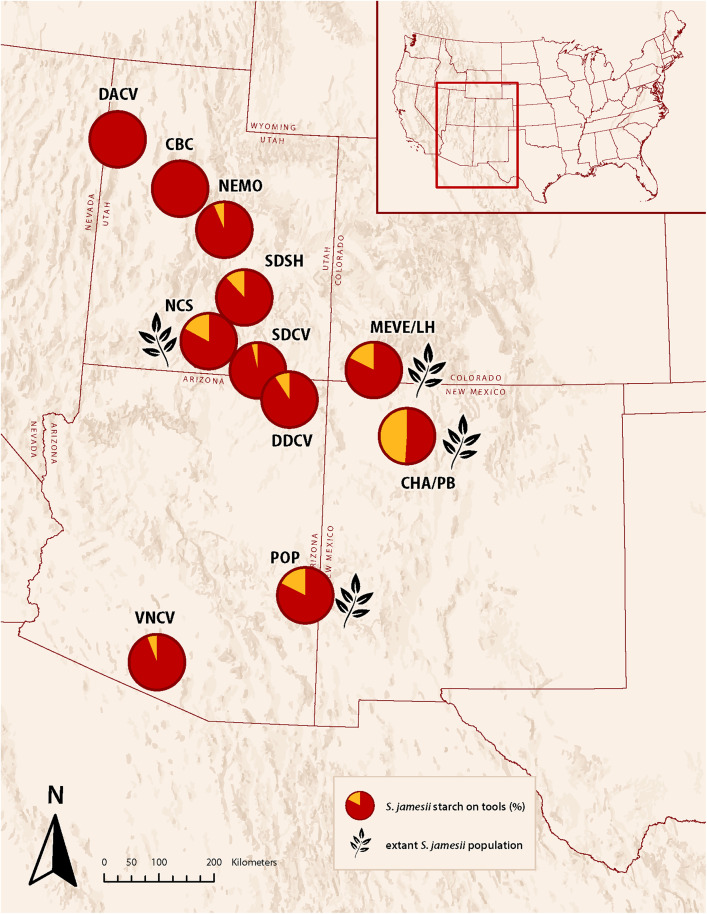
*S. jamesii* starch granule ubiquity (%, shown in yellow) compared to all other starch granules (red) on ground stone tools from archaeological sites beyond and within the boundaries of the *S. jamesii* range. Sites with ground stone tools that produced fewer than 100 granules are not shown on map due to small sample size. Pie charts with a potato leaf next to it indicate an extant potato population growing adjacent to site. (Basemap is the intellectual property of Esri and is used herein with permission. Copyright © 2025 Esri and its licensors. All rights reserved).

*S. jamesii* starch granules were found on tools at nine archaeological sites, seven to the north, one within and one to the south of the Mogollon region (Fig 4). Ubiquity values, however, varied greatly among the sites. The highest values (18–50%) were associated with ground stone tools from three sites in the Four Corners region of southern Utah, southwest Colorado, and northwest New Mexico (North Creek Shelter, Long House/Mesa Verde, Pueblo Bonito/Chaco Canyon). These sites also have one or more extant populations of *S. jamesii* growing nearby (Table 1). Farther to the north and south, ubiquity values decline (0–12%) with the exception of Point of Pines, which has a value of 18% along with an extant *S. jamesii* population. Ground stone tools from Cowboy Cave, Old Man Cave, and Gila Cliff Dwellings yielded no *S. jamesii* granules, but total granule counts were very low (n < 90). Sites well beyond the documented range of the species (Danger Cave, Camels Back Cave, Nephi Mounds, Sudden Shelter, Ventana Cave) also yielded none or few *S. jamesii* granules with very low ubiquity despite having high granule yields of other plant species (Table 2).

### Elder interviews

Of the 15 interviewees, 13 had specific knowledge of the Four Corners potato during their lives, either directly experienced or from communications with others (often grandparents). Only five, however, had indicated they had eaten it themselves (four females and one male) and all mentioned the practice of using *glésh* (special white clay) to reduce bitterness. All refer to the potato in terms of personhood as “*nímasii yázhí*” known as the tiny potato relative. Two, one male and one female, knew the species as a medicine for stomach aches and also prepared specifically for offerings to “more-than-human” beings and served with other spring foods at significant cultural gatherings. Elders noted that all root plants have significant knowledge and purpose interwoven with ancestral teachings by way of creation narratives. Land access, climate, and environment conditions have been challenges with tending to the potato “relatives” in the vast rugged region of the Colorado Plateau.

There was a striking difference in the way interviewees referred to the Four Corners potato; females tended to speak about it in the present tense and often possessed knowledge about how it was processed or eaten. Males, on the other hand, seemed to place the potato more in the past without specific knowledge of how to prepare it. Females also frequently mentioned specific places it was known to grow in the region (including Bears Ears (UT), Black Mesa (AZ), Dennehotso (AZ)). It was referred to by two women as “anywhere potato” that grows in very specific places, which we interpret to mean geographically widespread but a habitat specialist.

In addition to information about the potato, interviewees also gave detailed descriptions of other wild plants and their uses, mentioning 6–11 other species (especially wild onions, *Allium* spp.) and 2–7 domesticated crops. Interviewees also spoke of contemporary struggles across boundaries that render the social reproduction of traditional land-use practices and the mobility of traditional food systems that were often shaped by matrilineal societies.

### Nutritional analysis

Compared to organic red potatoes, tubers of *S. jamesii* have three times the protein, twice the calories, total carbohydrate, total sugar, calcium, phosphorus, magnesium, manganese, iron, B1, and are superior in terms of dietary fiber (Table 3). Sodium, niacin, and folate content were lower.

**Table 3:** *S. jamesii* nutritional data.

			Food value						
	calories	fat	saturated fat			CHO	dietary fiber	total sugars	protein
	kcal/100 g	g/100 g	g/100 g			g/100 g	g/100 g	g/100 g	g/100 g
*Solanum jamesii*: mean	131.37	0.14	0.03			26.86	4.37	1.49	5.68
SD	10.62	0.05	0.01			2.19	0.21	0.43	1.12
organic red potato	53.7	0.14	0.03			11.31	2.71	0.67	1.80
			**Mineral content**						
	Ca	P	K	Mg		Mn	Zn	Fe	Na
	Ppm	ppm	Ppm	ppm		ppm	ppm	ppm	ppm
*Solanum jamesii*: mean	61	1248	6758	606		3.0	13	16	93
SD	64	245	1231	112		0.7	4	5	61
organic red potato	33	500	5680	270		1.1	2.3	10	220
		**Vitamins**							
		niacin	total folate		B1-Thiamine		B2-Riboflav	vitamin D	
		mg/100 g	mg/100 g		mg/100 g		mg/100 g	mg/100 g	
*Solanum jamesii*: mean		2.21	0.0170		0.228		<.1	0	
SD		0.52	0.0073		0.014				
organic red potato		3.94	0.0590		0.115		<.1	0	

Food value, mineral content, and vitamins in greenhouse-grown tubers of *S. jamesii* compared to organic red potatoes.

## Discussion

Starch granules of *S. jamesii* were found on ground stone tools at nine archaeological sites, seven well to the north of the Mogollon Region in southern Utah, southwest Colorado, northwest New Mexico and northern Arizona. Three of these had high ubiquity (>18%, Fig 4), suggesting consistent, if not intensive use of the potato, through time. Only one site on the Mogollon Rim had a high ubiquity value (Point of Pines) and granules were few or absent from tools everywhere else examined. This suggests a cultural geography of tuber processing confined to a relatively narrow band of the northernmost Colorado Plateau, in what is now known as the Four Corners. The plant itself is present as both large and small populations, often within meters of the archaeological features that define these iconic sites [19].

North Creek Shelter is the oldest known archaeological site on the Colorado Plateau, having 30 cultural strata dating from 11,300 cal B.P [57]. *S. jamesii* granules are present as early as 10,900 cal B.P. and through the early and middle Holocene [17]. The extant population is downslope approximately 150 meters away. Genetic data clearly show that this population originated on the Mogollon Rim and is genetically identical to populations in the Bears Ears region of southeasten Utah, some 170 km away. However, other subpopulations in this Escalante valley only 4 km away originated from a different genetic source, indicating multiple, ancient introductions of tubers from vastly different source populations [9]. All occur on floodplains and under the shade of extensive groves of Gambel oak (*Quercus gambelii*) associated with abandoned fields and stone granaries. So abundant was this species in the local landscape (prior to overgrazing and development) that cavalry men in the 1860’s named Escalante “Potato Valley” and survived by eating the tubers [43].

Ground stone tools from Long House kivas and habitation rooms (one of the largest cliff dwellings at Mesa Verde National Park) dated to Pueblo III period (AD 1200 – AD 1300) and produced nine granules of *S. jamesii.* Although these were not very abundant relative to the entire starch assemblage (dominated by Poaceae, including *Z. mays* and Triticeae), ubiquity was relatively high. Below Long House, in both Spruce and Navajo canyons, are extensive subpopulations of *S. jamesii*. These occur in shade among trees and shrubs or along a shallow drainage that meanders across a floodplain. Ancient check dams are abundant, often holding lens of soil that support clusters of plants [16]. Other subpopulations are distributed near small drainages or at higher elevations in Mesa Verde where previous genetic studies found higher levels of genetic diversity. The overall complex of genetically diverse *S. jamesii* subpopulations at Mesa Verde has been termed a “mega-population” [32] that may have resulted from multiple founder events over time with unique leaf and tuber characteristics [9].

Pueblo Bonito is one of the oldest, largest, and most extensively excavated great houses at Chaco Canyon National Monument [58]. About half of the tools examined (ubiquity = 49%) yielded 68 starch granules assigned to *S. jamesii*. Some of these tools were excavated from rooms that show evidence of ritual use. Yarnell [18, p. 667] noted that *S. jamesii* tubers were “…found at Chaco Canyon in a mortuary bowl interred in Pueblo Bonito ruin.” These rooms include 28, 28-A, 33, 38, 39-B, and 80 where exotic and/or symbolic objects, such as cylindrical vessels with cacao residues, Scarlet Macaw feathers, turquoise beads, and *Olivella* shells were found [7,59,60]. Areas of intensive agriculture are found throughout Chaco and these often support large subpopulations of *S. jamesii* with earthen and stonework dams [16,61].

The use of *S. jamesii* at Point of Pines might be expected, given that the site is embedded within the vast, nearly continuous distribution of the species in the Mogollon region, far to the south of the Colorado Plateau. Presumably, it was not cultivated here, only collected from the wild and processed along with maize. Then it is somewhat surprising that no evidence of potato use was found on ground stone at Gila Cliff Dwellings, which also has access to these abundant and widespread Mogollon populations.

**Fig 5:**
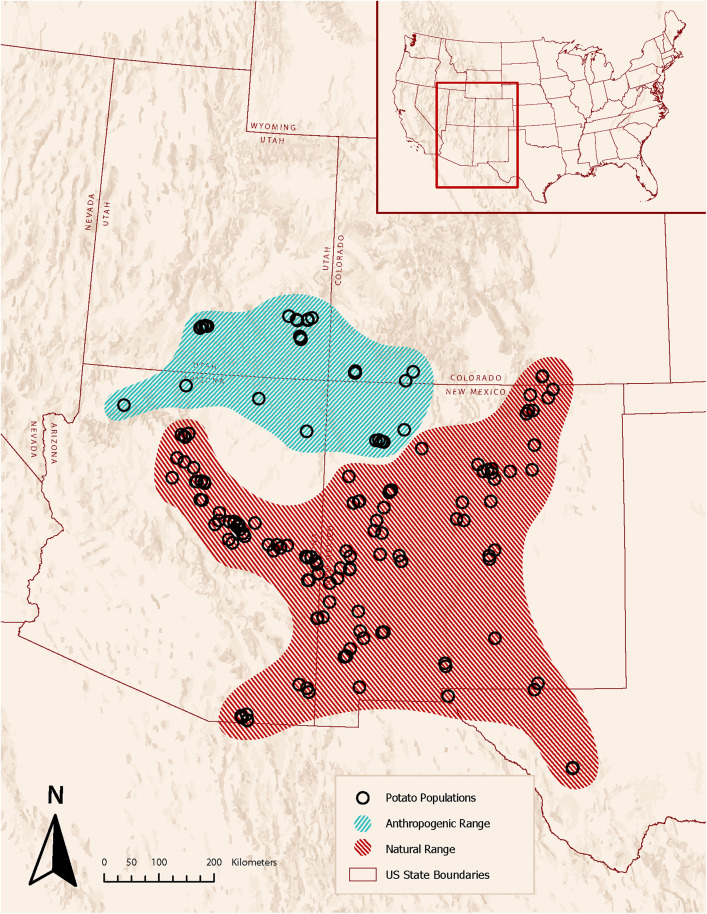
Proposed delineation of the anthropogenic range of *S. jamesii* (blue) based upon starch granule and genetic data. Most plant populations in this portion of the range are associated with one or more archaeological sites and are of human origin. (Basemap is the intellectual property of Esri and is used herein with permission. Copyright © 2025 Esri and its licensors. All rights reserved).

Patterns of *S. jamesii* use, as evidenced by starch granules on ground stone tools, corroborate previously established genetic patterns from extant populations of the species that proved long-distance transport of tubers by people in the deep past [9]. Plants growing at North Creek Shelter were established from tubers with alleles that originated in the Mogollon region, some 400 km to the south. Populations at Mesa Verde were established from a tuber source that extends into northwest Texas, while populations at Chaco Canyon have a mix of alleles from totally different sources. These and other genetically surveyed populations associated with archaeological sites in the Four Corners region were shown to have undergone a severe bottleneck due to human-caused founder effect, rendering them unable to sexually reproduce and disperse on their own [14]. Consequently, we propose that the documented distribution of *S. jamesii* is an amalgam of anthropogenic and natural populations, the former in a relatively narrow band on the northern Colorado Plateau in the Four Corners region and the latter widely scattered along the broad crescent of the Mogollon Plateau and through the uplands of southern Arizona and southern New Mexico (Fig 5).

Perhaps most importantly, the interviews we conducted reveal that Diné farmers and elders still know, grow, and eat *S. jamesii* tubers, as well as use them for spiritual purposes, such as in the blessing way, water offering, and seedling ceremonies. The Puebloan also still eat *S. jamesii* tubers and recall that “It was the best snack for social dances in the kiva and for ceremonies between ditch cleaning and before planting.” [62]. Efforts are underway to ensure Indigenous farmers have access to tubers from traditional, undisturbed populations throughout the range of the species [63].

In western North America the natural distributions of plant and animal species have largely determined the availability of food resources for ancient foragers. Local transport of foods, most often less than 50 km distance [6], seem to be evident throughout most of the Holocene archaeological record, owing to the general abundance of food in the landscape (see [64] for an extensive plant list from the Pacific Northwest). Evidence for long-distance movement of foods, however, is less common. The costs of long-distance transport have often been shown to outweigh the benefits, especially with foods that are calorie-poor, difficult to transport, and readily spoil in the process [65–69]. High moisture content, processing requirements, bulkiness, nutritional degradation, and consumption by travelers diminish utility and value over extended distances and prolonged journeys [6,68,70,71]. This theoretical approach may, in some cases, underestimate the occurrence of long-distance transport and dispersal of plants by humans.

High-value resources with spiritual, social, political or medicinal properties (“elite” *sensu* [72]) and, as well as having food potential might have been an exception [8,66,67,70,73,74]. Turquoise, obsidian, salt, cacao, Scarlet Macaws (and their feathers), copper bells, ceramics, blankets, and *Olivella* shell beads [7,59,75–79], are known to have been exchanged among groups in western North America and the Southwest over distances exceeding 1000 km. Furthermore, there is strong evidence for the migration of people across the Southwest based on the presence of extensive Chacoan roads [61,80,81] and social networks [82]. We find that tubers of *S. jamesii* joined those processions many times, over long distances and difficult terrain.

Having established the ancient use and transport of *S. jamesii* using multiple forms of evidence, the question of whether this species was on a domestication trajectory must be addressed by directly linking human activities to changes in plant structure and function [83]. Such markers may take the form of unique morphological/physiological characteristics in one or more plant populations (e.g., increase in seed/fruit size, a reduction in seed and/or fruit coat thickness,), alterations of genetic composition (e.g., reduced allelic diversity), or demographic restructuring (e.g., smaller, isolated populations with impaired sexual reproduction). These indicators of human manipulation have been noted in *S. jamesii* from archaeological sites, including variations in leaf morphology [9], freezing tolerance [84], extended tuber dormancy [15], sprouting resilience [85], and lack of seed and fruit production [14], and we suspect there are other such traits. In the Southwestern USA, there are some promising lines of research that suggest domestication of native plants, such as amaranth [4] and barley [3], but the evidence often falls short when compared to what is known about domesticates from other regions of the world [26,86–93]. Increased confidence in detecting plant domestication comes from using multiple, independent lines of evidence, including archaeological, genetic, life history, ecological, biogeographical, ethnographic and linguistic [64,91]. More recently, the combination of genetic and morphological analyses on remnant populations of several species of *Agave* strongly suggests a domestication trajectory in Arizona [2]. Similar genetic analysis has been used on hazelnut (*Corylus cornuta*) in the Pacific Northwest [94].

The archaeological evidence presented here supports the ancient use and long-distance transport of *S. jamesii* tubers. Some of those tubers established cultivated populations that persist to this day within an anthropogenic range that defines a cultural geography centered around this species. Along with genetic [9,32] and demographic [14,19] data from populations associated with archaeological sites, the totality of the evidence firmly establishes the initial stages of *S. jamesii* domestication by Indigenous people took place across the Colorado Plateau, USA.

## Supporting information

S1 Table Ground stone tools analyzed in this study. Site name, repository, catalog number, lab specimen number, and provenience of each ground stone tool analyzed for starch.
(XLSX)

S2 Table Starch granule measurements. Lengths and morphological characters measured and documented on each starch granule from all ground stone tools (n = 401) analyzed in this study. Each tab contains starch granule data from an archaeological site (n = 14). All starch granules have length measurements, but not all morphological characters were documented on all granules.
(XLSX)

S3 Table Starch granules assigned to *S. jamesii.* Taxonomic assignment of granules to *S. jamesii* is based on the possession of an eccentric hilum and two or more diagnostic characters.
(XLSX)
